# Pseudoginsenoside F11, a Novel Partial PPAR**γ** Agonist, Promotes Adiponectin Oligomerization and Secretion in 3T3-L1 Adipocytes

**DOI:** 10.1155/2013/701017

**Published:** 2013-12-18

**Authors:** Guoyu Wu, Junyang Yi, Ling Liu, Pengcheng Wang, Zhijie Zhang, Zhen Li

**Affiliations:** ^1^MOE Key Laboratory of Bioinformatics, School of Life Sciences, Tsinghua University, Beijing 100084, China; ^2^Institute of Chinese Materia Medica, China Academy of Chinese Medical Sciences, Beijing 100700, China

## Abstract

PPAR**γ** is a nuclear hormone receptor that functions as a master regulator of adipocyte differentiation and development. Full PPAR**γ** agonists, such as the thiazolidinediones (TZDs), have been widely used to treat type 2 diabetes. However, they are characterized by undesirable side effects due to their strong agonist activities. Pseudoginsenoside F11 (p-F11) is an ocotillol-type ginsenoside isolated from *Panax quinquefolium L.* (American ginseng). In this study, we found that p-F11 activates PPAR**γ** with modest adipogenic activity. In addition, p-F11 promotes adiponectin oligomerization and secretion in 3T3-L1 adipocytes. We also found that p-F11 inhibits obesity-linked phosphorylation of PPAR**γ** at Ser-273 by Cdk5. Therefore, p-F11 is a novel partial PPAR**γ** agonist, which might have the potential to be developed as a new PPAR**γ**-targeted therapeutics for type 2 diabetes.

## 1. Introduction

The nuclear hormone receptor PPAR*γ* (peroxisome proliferator-activated receptor *γ*) is a ligand-activated transcription factor highly expressed in the adipose tissues [1]. By binding to PPAR*γ*-responsive regulatory elements as heterodimers with retinoid X receptor (RXR), PPAR*γ* regulates the expression of networks of genes involved in adipogenesis, lipid metabolism, inflammation, and maintenance of metabolic homeostasis [2]. PPAR*γ* consists of an amino terminal activation domain (AF-1), a highly conserved DNA-binding domain (DBD), and a C-terminal ligand-binding domain (LBD) containing a ligand-dependent transactivation domain (AF-2) [3]. Ligand binding promotes a conformational change which allows for differential recruitment of cofactors and subsequent modulation of PPAR*γ* activity [4, 5].

PPAR*γ* is the pharmacological target of the insulin-sensitizing thiazolidinediones (TZDs) that have been widely used in the treatment of type 2 diabetes. TZDs function as selective PPAR*γ* ligands and induce transcription of PPAR*γ*-targeted genes [6]. Derivatives of TZD, such as rosiglitazone (Avandia) and pioglitazone (Actos), are highly effective in treating type 2 diabetes and are well tolerated by the majority of patients [1]. However, they are associated with various undesirable side effects, including weight gain, fluid retention, edema, congestive heart failure, and bone fracture [7, 8]. Long-term use of TZDs may be associated with increased risk of bladder cancer [9]. These limitations have raised substantial concerns and significantly impaired their future in many countries [10]. Therefore, it is critical to develop TZD substitutes for improved therapies of type 2 diabetes. Studies in animal models and in clinical trials have shown that the side effects of TZD can be minimized without loss of insulin sensitization by partial PPAR*γ* agonists [8, 11–16].

Adiponectin is an insulin-sensitizing adipokine secreted specifically by the adipose tissues as high, medium, and low molecular weight forms (HMW, MMW, and LMW) [17, 18]. The HMW adiponectin is more metabolically active and has a more relevant role in insulin sensitivity and in protecting against diabetes [19–21]. The serum level of adiponectin inversely correlates with obesity and directly correlates with insulin sensitivity [22, 23]. Additionally, serum adiponectin levels increase with weight loss, caloric restriction, or TZD treatment [24–27]. The PPAR*γ* agonists increase serum levels of adiponectin by upregulating the transcription of adiponectin through a PPAR*γ*-responsive element (PPRE) present in the promoter of adiponectin [26, 28]. In addition, PPAR*γ* agonists regulate adiponectin oligomerization and secretion by increasing the expression of Ero1-L*α* and DsbA-L, which have been reported to promote adiponectin oligomerization and secretion [29, 30].

Pseudoginsenoside F11 (p-F11) is an ocotillol-type ginsenoside isolated from the roots and leaves of *Panax quinquefolium L.* (American ginseng) [31–33]. p-F11 has been demonstrated to antagonize the learning and memory deficits induced by morphine, scopolamine, and methamphetamine [34–37], suggesting that p-F11 might be a candidate for the treatment of drug abuse. With its antiamnesic effect, p-F11 might also serve as a potential therapeutic target for Alzheimer's disease [38].

p-F11 was identified as one of the natural compounds that can promote preadipocyte differentiation in our screen for partial PPAR*γ* agonists. In the present study, we further examined the effect of p-F11 on adipogenesis and the transcriptional activity of PPAR*γ*. We demonstrate that p-F11 is a novel partial PPAR*γ* agonist. In addition, we found that p-F11 inhibits Cdk5-mediated phosphorylation of PPAR*γ* and promotes the oligomerization and secretion of adiponectin. Thus, p-F11 is a potential PPAR*γ*-targeted drug for the treatment of type 2 diabetes.

## 2. Materials and Methods

### 2.1. Materials

Pseudoginsenoside F11 (p-F11), rosiglitazone, GW9662, 3-isobutyl-1-methylxanthine, dexamethasone, insulin, Oil Red O, and antibody against *β*-actin were purchased from Sigma. PPAR*γ* antibody and phospho-(Ser) CDKs substrate rabbit monoclonal antibody were purchased from Santa Cruz or Cell Signaling, respectively. TNF-*α* was purchased from Sino Biological.

### 2.2. RNA Isolation and Real-Time PCR

Total RNA was isolated from 3T3-L1 adipocytes and quantitative real-time PCR was used as described previously [39]. PCR reactions were carried out in an ABI PRISM 7500 real-time PCR system. The expression levels of adiponectin and PPAR*γ* were normalized using *β*-actin as an internal control.

### 2.3. Cell Culture, Cell Differentiation, and Treatment

3T3-L1 preadipocytes (ATCC) and 293T cells were grown in DMEM (Invitrogen) supplemented with 10% FBS (Hyclone) at 37°C in 5% CO_2_ and 3T3-L1 preadipocytes were induced to differentiate into mature adipocytes by the standard protocol as previously described [39, 40]. In order to examine the effect on differentiation, p-F11 or rosiglitazone was added to the culture medium in the presence or absence of GW9662 during differentiation. On Day 8 after differentiation, the lipid droplets in the cells were stained and quantified as previously described [40].

To examine the effect of p-F11 on adiponectin oligomerization and secretion, mature 3T3-L1 adipocytes were starved in a serum-free medium containing 0.05% BSA for 24 h, followed by treatment with p-F11 for another 24 h.

To examine the effect on PPAR*γ* phosphorylation at Ser-273, 3T3-L1 adipocytes were pretreated with TNF-*α*, followed by treatment with p-F11 or rosiglitazone for 1 hour, as described previously [11].

### 2.4. SDS-PAGE and Western Blotting Analysis

Cell lysates or culture medium of 3T3-L1 adipocytes were subjected to 2–15% gradient gel electrophoresis under nonreducing and nonheat-denaturing conditions as described previously [17, 39, 40]. Adiponectin oligomers and the total amount of adiponectin were detected using antibodies against the globular domain or the N-terminal peptide of adiponectin. PPAR*γ* was detected using antibodies specific for PPAR*γ*, and phosphorylated PPAR*γ* at Ser-273 was detected using anti-CDK substrate antibody after immunoprecipitation with anti-PPAR*γ* antibody [11]. The amount of p-PPAR*γ*, total PPAR*γ*, total adiponectin, and adiponectin oligomers was quantified by analyzing the bands on the western blots using NIH ImageJ software. All experiments were performed at least three times and representative results were presented. Results are expressed as the means ± SD. Student's *t*-test was used for statistical analyses; *P* value < 0.05 was considered to be statistically significant.

### 2.5. Luciferase Reporter Assay

293T cells were transfected with PPRE-TK-Luciferase reporter along with PPAR*γ* and RXR*α* expression vectors. 24 hours after transfection, the cells were treated with p-F11 or rosiglitazone in the presence or absence of GW9662. The cells were harvested after treatment for 24 hours. A reporter luciferase assay kit (Promega) was used to measure luciferase activity according to the manufacturer's instructions. Luciferase activities were normalized to Renilla activities cotransfected as an internal control [40].

## 3. Results

### 3.1. p-F11 Is a Partial PPAR*γ* Agonist with Modest Adipogenic Activity

To examine the effect of p-F11 on differentiation, 3T3-L1 preadipocytes were induced to differentiate in the presence of p-F11. Rosiglitazone (Rosi), which was reported to activate PPAR*γ* and promote preadipocyte differentiation [6], was used as a positive control in all of our experiments.

**Figure 1:**
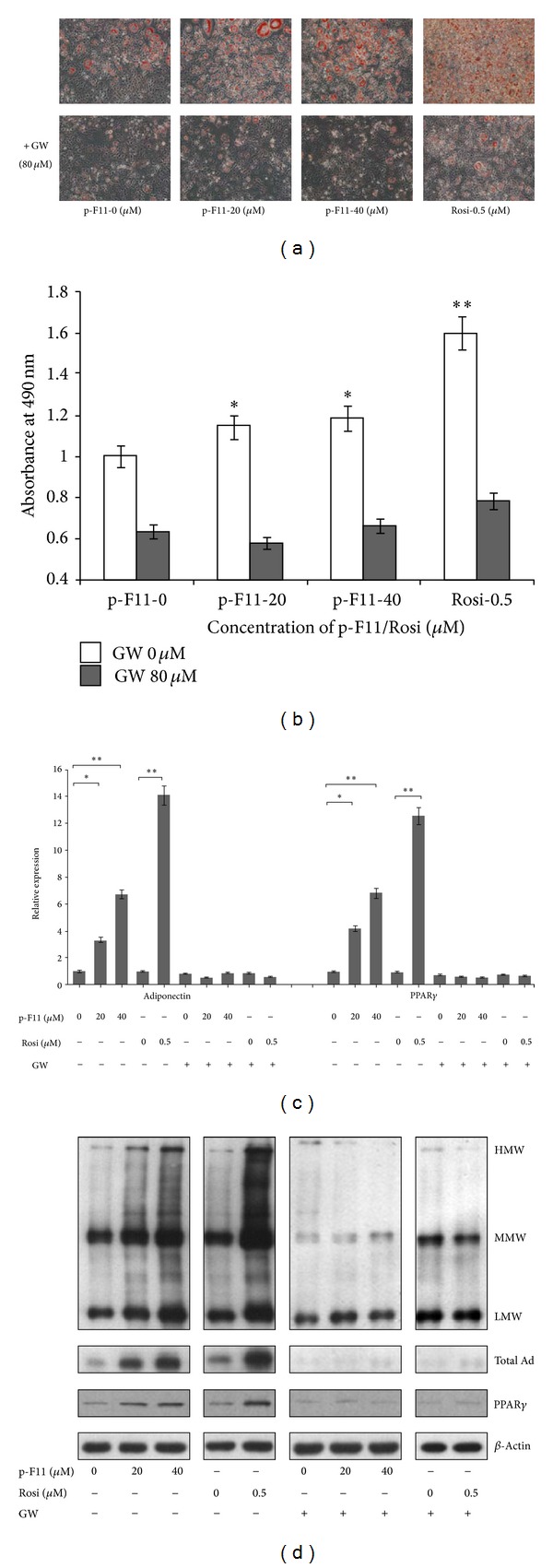
p-F11 promotes preadipocyte differentiation by activating PPAR*γ*. 3T3-L1 preadipocytes were induced to differentiate with 0, 20, 40 *μ*M Pseudoginsenoside F11 (p-F11) or 0.5 *μ*M rosiglitazone (Rosi) in the absence or presence of 20 *μ*M GW9662 for 8 days. (a) The cells were stained with Oil Red O. Quantification of the staining results was presented in (b). Results (mean ± SD, *n* = 3) were expressed as a percentage of the control (p-F11 0 *μ*M). *P < 0.05; **P < 0.01. (c) The mRNA level of adiponectin and PPAR*γ* was examined by quantitative real-time PCR. The results were expressed relative to *β*-actin and presented as a percentage of the control. (d) The cell lysates were subjected to 2%–15% gradient gel electrophoresis under nonreducing and non-heat-denaturing conditions to detect the three oligomeric forms of adiponectin (LMW, MMW, and HMW) (top panel). The amount of total adiponectin (Total Ad), PPAR*γ*, or actin was detected with antibodies against the N-terminal peptide of adiponectin, PPAR*γ*, or *β*-actin, respectively.

We found that p-F11 promoted the differentiation of 3T3-L1 preadipocytes. The number of lipid droplets, evaluated by Oil Red O staining, was increased by p-F11 in a dose-dependent way (Figure 1(a)). However, 40 *μ*M of p-F11 induced adipogenesis to a lesser extent than 0.5 *μ*M Rosi (Figure 1(b)), indicating that p-F11 is less potent than Rosi in promoting adipogenesis.

PPAR*γ* is a dominant regulator of adipocyte differentiation [41]. To determine whether PPAR*γ* is involved in p-F11-promoted differentiation, 3T3-L1 preadipocytes were induced to differentiate in the presence of both p-F11 and GW9662, a specific PPAR*γ* antagonist. We found that the effect of p-F11 on differentiation was completely abolished by GW9662 (Figures 1(a) and 1(b)). This result suggested that p-F11 promotes differentiation by activating PPAR*γ*.

To further investigate the effect of p-F11 on differentiation, we examined the expression of PPAR*γ* as well as adiponectin, which is a PPAR*γ*-responsive gene. Similar to Rosi, p-F11 increased the mRNA and protein level of both PPAR*γ* and adiponectin (Figures 1(c) and 1(d)). In addition, the level of different adiponectin oligomers (LMW, MMW, and HMW) was increased by p-F11 dose dependently (Figure 1(d)). GW9662 blocked the effects of p-F11 or Rosi (Figures 1(c) and 1(d)). Therefore, p-F11 upregulates the expression and oligomerization of adiponectin by activating PPAR*γ* during the differentiation of 3T3-L1 preadipocytes.

### 3.2. p-F11 Promotes Adiponectin Oligomerization and Secretion in 3T3-L1 Adipocytes

**Figure 2:**
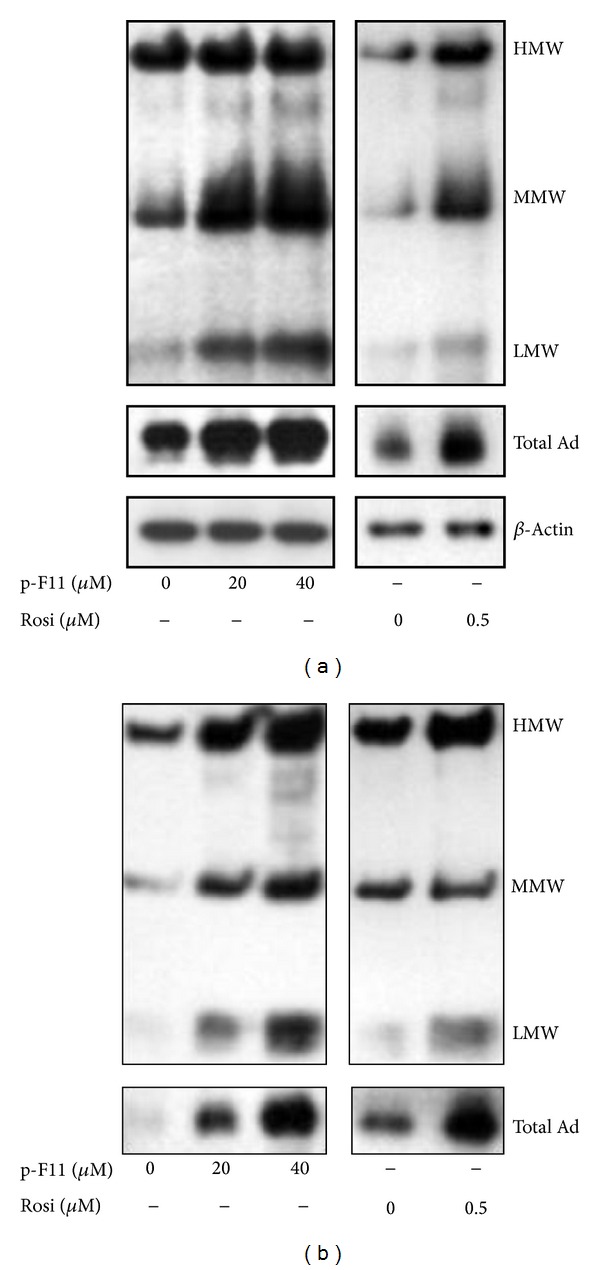
p-F11 promotes adiponectin oligomerization and secretion in 3T3-L1 adipocytes. 3T3-L1 adipocytes were treated with p-F11 or Rosi for 24 hours. The amount of each oligomer and total adiponectin in the cell lysates (a) or in the culture medium (b) were detected as described in Figure 1(d).

To examine the effect of p-F11 on adiponectin in mature adipocytes, we treated 3T3-L1 adipocytes with p-F11 or rosiglitazone for 24 hours. The cellular level of adiponectin oligomers was increased by p-F11 in a dose-dependent way (Figure 2(a)). Furthermore, p-F11 increased the secretion of different adiponectin oligomers, particularly the HMW adiponectin, in a way similar to rosiglitazone (Figure 2(b)). These results suggested that p-F11 promotes the oligomerization and secretion of adiponectin in mature adipocytes.

### 3.3. p-F11 Exhibits PPAR*γ*-Activating Activity in 293T Cells

**Figure 3:**
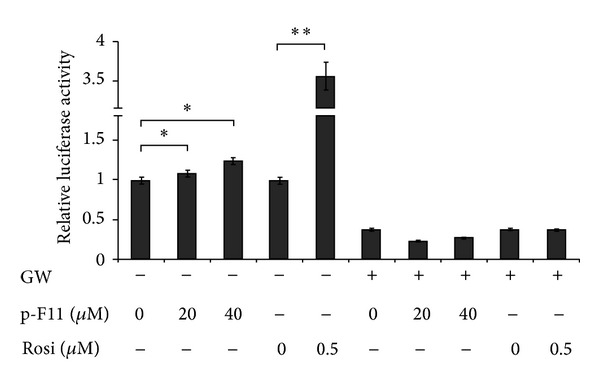
p-F11 exhibits PPAR*γ*-activating activities in 293T cells. 293T cells were transfected with PPRE-TK-Luciferase reporter along with PPAR*γ* and RXR*α* expression vectors for 24 hours. The cells were treated with p-F11 or Rosi in the presence or absence of GW9662 for another 24 hours. The cell extracts were subjected to luciferase assay. The results were presented as a percentage of the control (p-F11 0 *μ*M).

To further demonstrate the PPAR*γ*-activating activity of p-F11, we examined the effect of p-F11 on PPAR*γ* transcriptional activity by luciferase reporter assays. 293T cells were transfected with PPRE-TK-Luciferase reporter along with PPAR*γ* and RXR*α* expression vectors, followed by treatment with p-F11 for 24 hours. p-F11 dose dependently increased the transcriptional activity of PPAR*γ*, which was abrogated by GW9662 (Figure 3). However, p-F11 exhibited lower transcriptional activity than Rosi. These results further demonstrated that p-F11 is a partial PPAR*γ* agonist.

### 3.4. p-F11 Inhibits Obesity-Linked Phosphorylation of PPAR*γ* at Ser-273 in 3T3-L1 Adipocytes

In addition to their capacity to enhance the transcriptional activity of PPAR*γ*, PPAR*γ* agonists have a separable biochemical activity, blocking the obesity-linked phosphorylation of PPAR*γ* at Ser-273 by Cdk5. This phosphorylation results in the dysregulation of a subset of beneficial PPAR*γ*-regulated genes, such as adiponectin, that are known to be associated with insulin sensitization [11]. The insulin-sensitizing effects of PPAR*γ* agonists are more closely correlated with their ability to inhibit phosphorylation of PPAR*γ* at Ser-273 [12–14].

**Figure 4:**
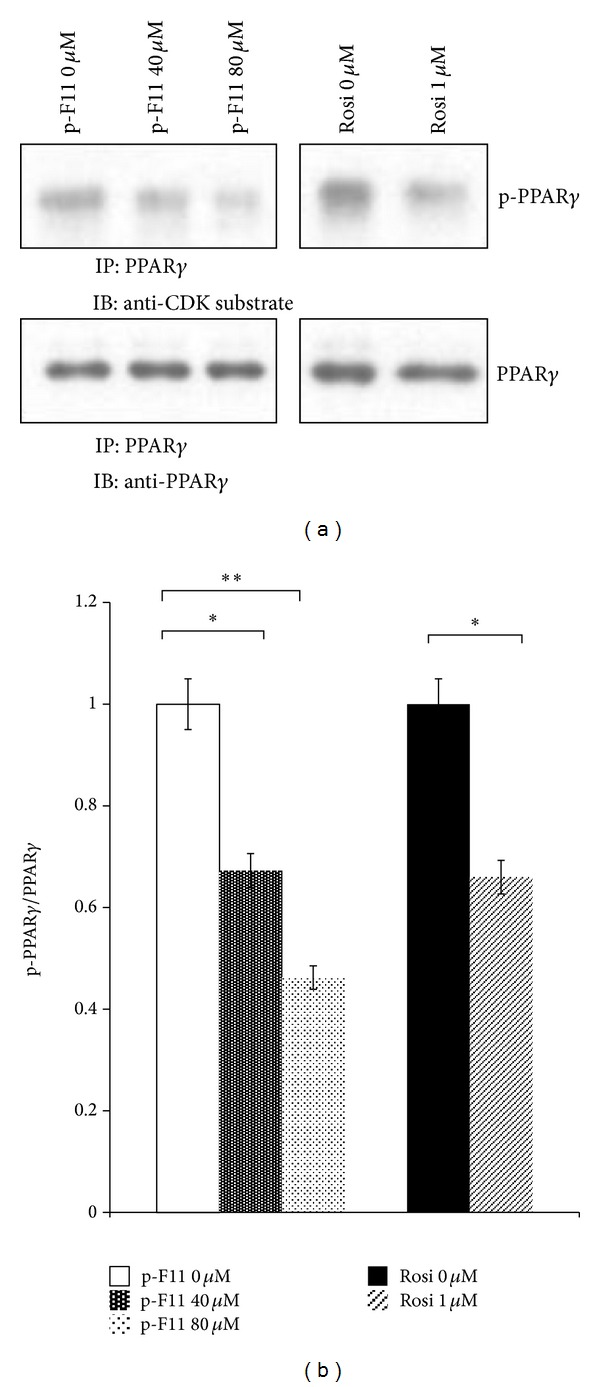
p-F11 inhibits PPAR*γ* phosphorylation at Ser-273. 3T3-L1 adipocytes were pretreated with TNF-*α*, followed by treatment with p-F11 or Rosi for 1 hour. Phosphorylated PPAR*γ* at Ser-273 (p-PPAR*γ*) was detected using anti-CDK substrate antibody after immunoprecipitation with anti-PPAR*γ* antibody. The amounts of p-PPAR*γ* and total PPAR*γ* were quantified and p-PPAR*γ*/PPAR*γ* ratio was presented as a percentage of the control (p-F11 0 *μ*M or Rosi 0 *μ*M).

To examine the effect of p-F11 on Ser-273 phosphorylation of PPAR*γ*, 3T3-L1 adipocytes were first treated with TNF-*α* to induce the phosphorylation, followed by treatment with p-F11 or Rosi. We found that p-F11 dose dependently decreased Ser-273 phosphorylation of PPAR*γ* (Figure 4(a)). Approximately 50% inhibition was seen with 80 *μ*M p-F11 (Figure 4(b)). Therefore, p-F11 inhibits phosphorylation of PPAR*γ* at Ser-273.

## 4. Discussion

In this study, we found that p-F11 promoted the differentiation of 3T3-L1 preadipocytes, which was completely inhibited by GW9662 (Figure 1). This result suggested that p-F11 promoted adipogenesis by activating PPAR*γ*. We also found that p-F11 promoted adiponectin oligomerization and secretion in 3T3-L1 adipocytes (Figure 2). Furthermore, p-F11 activated the transcriptional activity of PPAR*γ* in the reporter assay (Figure 3). Therefore, PPAR*γ* is a novel PPAR*γ* agonist. However, 40 *μ*M of p-F11 elicited weaker adipogenic and transcriptional activity than 0.5 *μ*M of Rosi (Figures 1 and 3). Therefore, p-F11 is a partial PPAR*γ* agonist.

Like other PPARs, PPAR*γ* has a large ligand binding pocket which allows it to accommodate a wide range of ligands, including endogenous ligands such as native and modified fatty acids and prostaglandins [42]. Ligand binding induces a large conformational change in helix 12 of LBD of PPAR*γ*, which creates a hydrophobic cleft on the surface of the proteins that serves as a high affinity docking site to recruit transcriptional coactivators [4]. The partial agonist activity of GQ-16 results from its weak ability to stabilize helix 12 of PPAR*γ*, which is different from the binding mode of TZDs [12]. INT131, another partial PPAR*γ* agonist with robust glucose-lowering activity and reduced side effects, forms hydrophobic contacts with the ligand-binding pocket without direct hydrogen-bonding interactions to key residues in helix 12 that are characteristic of full agonists [43]. With its unique structure, it remains to be determined how p-F11 interacts with PPAR*γ*.

Ser-273 is situated immediately adjacent to the first *β*-sheet of PPAR*γ*, which has been shown to mediate contacts between PPAR*γ* and RXR*α*. Phosphorylation of Ser-273 by Cdk5 disrupts the contacts, leading to decreased expression of a subset of PPAR*γ*-regulated genes which are known to be associated with insulin sensitization [11]. Therefore, Ser-273 phosphorylation is a key determinant of whole body insulin sensitivity [11, 44, 45]. TZDs, such as rosiglitazone and pioglitazone, are synthetic ligands that function as strong agonists on PPAR*γ* and potent insulin sensitizers. However, they have several undesirable side effects, such as weight gain and edema. New classes of antidiabetic drugs can be developed by specifically targeting the Cdk5-mediated phosphorylation of PPAR*γ* at Ser-273. Several PPAR*γ* ligands, with or without classical agonist properties, were identified so far. GQ-16, MRL24, and amorfrutins are partial PPAR*γ* agonists, whereas SR1664 lacks classical transcriptional agonism. They all inhibit Ser-273 phosphorylation of PPAR*γ* [11–14]. GQ-16 has been shown to do so by stabilizing the helix 3 and *β*-sheet region of PPAR*γ*, shielding Ser-273 from phosphorylation by Cdk5 [12]. All these agonists promote insulin sensitization without weight gain and other some unwanted side effects, demonstrating that the insulin-sensitizing effects of these PPAR*γ* ligands are derived from their capacity to inhibit phosphorylation. In other words, the undesirable side effects of TZD drugs might be due to their strong agonist actions. Therefore, moderate activation of PPAR*γ* might be better to uncouple the insulin-sensitizing effects from the adverse side effects. p-F11 exhibits weak transcriptional activity compared to rosiglitazone (Figures 1 and 3). On the other hand, p-F11 is very potent in blocking Cdk5-mediated phosphorylation of PPAR*γ*, with higher concentrations being as efficacious as Rosi (Figure 4). p-F11 also promotes adiponectin oligomerization and secretion (Figure 2). We are currently examining the effect of p-F11 on the insulin sensitivity of diabetic mice.

Asian ginseng (*Panax ginseng*) and American ginseng (*Panax quinquefolium L.*) are perennial aromatic herbs that are widely used in oriental medicine and have been demonstrated to have various health benefits including diabetes treatment. For example, American ginseng has been shown to be effective in improving glycemic control in type 2 diabetes [46]. Only present in American ginseng, p-F11 has been used for the unambiguous identification of Asian ginseng and American ginseng [32, 33]. p-F11 has been reported to exhibit neuroprotective and antiamnesic effects [34–37]. In this paper, we demonstrated that p-F11 is a partial PPAR*γ* agonist, which is the first natural compound in American ginseng to have this activity. p-F11 does not seem to have any toxic effects on cell viability during differentiation or in mature adipocytes (data not shown). The fact that p-F11 exhibits partial efficacy in activating PPAR*γ*, but increases adiponectin secretion and inhibits obesity-linked phosphorylation of PPAR*γ*, makes it a potential therapeutic agent for the treatment of type 2 diabetes.
